# Emotion-related impulsivity moderates the cognitive interference effect of smartphone availability on working memory

**DOI:** 10.1038/s41598-019-54911-7

**Published:** 2019-12-06

**Authors:** Natale Canale, Alessio Vieno, Mattia Doro, Erika Rosa Mineo, Claudia Marino, Joël Billieux

**Affiliations:** 1 Department of Developmental and Social Psychology, University of Padova, Padova, Italy; 2 Addictive and Compulsive Behaviours Lab, Institute for Health and Behaviour, University of Luxembourg, Esch-sur-Alzette, Luxembourg; 3 Institute of Psychology, University of Lausanne, Lausanne, Switzerland

**Keywords:** Human behaviour, Risk factors

## Abstract

Although recent studies suggest that the mere presence of a smartphone might negatively impact on working memory capacity, fluid intelligence, and attentional processes, less is known about the individual differences that are liable to moderate this cognitive interference effect. This study tested whether individual differences in emotion-related impulsivity traits (positive urgency and negative urgency) moderate the effect of smartphone availability on cognitive performance. We designed an experiment in which 132 college students (age 18–25 years) completed a laboratory task that assessed visual working memory capacity in three different conditions: two conditions differing in terms of smartphone availability (smartphone turned off and visible, smartphone in silent mode and visible) and a condition in which the smartphone was not available and was replaced by a calculator (control condition). Participants also completed self-reports that assessed their thoughts after the task performance, positive/negative urgency, and problematic smartphone use. The results showed that participants with higher positive urgency presented increased cognitive interference (reflected by poorer task performance) in the “silent-mode smartphone” condition compared with participants in the “turned-off smartphone” condition. The present study provides new insights into the psychological factors that explain how smartphone availability is liable to interfere with high-level cognitive processes.

## Introduction

Smartphones are now used worldwide as one of the main information and communication technologies, especially among college students who are digital natives, having used these devices from an early age. College students use smartphones to engage in a wide range of activities, such as communicating with their peers, using social networks, searching for information, or gaming^1^. Recent studies have shown that college students check their smartphone 60 times a day on average, with daily usage of more than 5 h^2–4^. Although this portable media device offers many advantages in terms of communication among systems and individuals for work and for leisure, a potential adverse effect of mobile phone use is its distractive power via, for example, constant notifications^5^. An increasing body of empirical research suggests that digital interruptions may harm well-being and mental health^6,7^. Frequent interruptions by smartphones have been found to be responsible for higher levels of inattention, which in turn predict lower productivity and psychological well-being longitudinally^7^.

## Smartphone-Related Interruptions

According to a recent review that focused on the effects of smartphone on cognition, two forms of smartphone-related interruptions can be distinguished: *endogenous* and *exogenous*^8^. Endogenous interruptions consist in drifts of attention from users’ thoughts to smartphone-related activities because of the urge to modify an internal state (such as experiencing boredom in the current task and consequently having a desire to engage in more rewarding activities), which can lead users to engage in using the smartphone for activities that are not related to the ongoing task. Previous studies suggested that the detrimental effect of the distraction increases together with the attractiveness of the smartphone-related content (i.e., image vs. text)^9,10^. In contrast, exogenous interruptions consist in drifts of attention from users’ thoughts to smartphone-related activities because of specific environmental cues, such as smartphone-related alerts and notifications, or smartphone-related cues such as seeing someone else using a smartphone, or even talking with someone else about a smartphone activity (such as text messages and emails, gaming, or using social network sites).

College students are likely to be vulnerable to experiencing negative consequences from these exogenous interruptions^11–14^. In fact, recent studies found that (i) smartphone use during class time and study time is distracting and diminishes information retention, (ii) the sound of an incoming message/notification or simply the presence of a smartphone can interrupt a college student’s ability to remain focused on a lecture or homework assignment, and (iii) the sound of a cell phone ringing during class and the fact of having a smartphone both negatively affect the performance of college students on a subsequent quiz related to the lecture^13–16^. In another study, Lee and colleagues found that individuals who were allowed to use their cell phones (e.g., for receiving text messages) performed worse on a multiple-choice test than did those students who did not have access to their smartphones^17^. Notably, smartphone-related interference occurs even when the user attempts to ignore it. For instance, receiving cell phone notifications (calls or text messages) was shown to significantly disrupt performance on a task requiring sustained attention, even when college students did not directly interact with the mobile device during the task^18^. Smartphones tend to automatically attract the attentional focus of individuals engaged in competing tasks for which smartphones are task irrelevant^19–21^.

Moreover, the mere presence of a smartphone (turned off) has been found to have a negative impact on working memory capacity, fluid intelligence, and attentional processes^21,22^. It has been proposed that this “cognitive interference effect” impairs the ability to voluntarily inhibit high-priority yet task-irrelevant habits such as checking a smartphone^19,21,23^. Two studies have provided data that support the cognitive interference effect of the mere presence/availability of a smartphone. First, Thorton and colleagues^22^ found that participants in a visually salient cell phone condition, compared with college students with a notebook placed in front of them, performed poorly on the most difficult parts of two neuropsychological tasks: the digit cancellation task (used to assess attention, cognitive capacity, and executive functioning^24,25^) and the Trail Making Test (a measure that requires a variety of abilities for successful performance, including attentional processes, mental flexibility, and motor function^26^). Second, Ward and colleagues^21^ conducted a series of studies in which participants were asked to complete two measures of domain-general cognitive function (available working memory capacity and fluid intelligence). In a first experiment, smartphone availability was manipulated by asking undergraduates to place their device (in silent mode): (i) nearby and in sight (desk), (ii) nearby and out of the sight (pocket/bag), and (iii) in a separate room (other room). Their data showed that the availability of the participants’ smartphones worsened performances on two tasks: the Automated Operation Span task (OSpan, a prominent measure of working memory capacity^27^) and a 10-item subset of Raven’s Standard Progressive Matrices (a nonverbal measure of fluid intelligence^28^). In a second experiment, availability was manipulated in terms of smartphone power (silent mode versus off) and location (as in the first experiment), and the potential effect of smartphone addiction symptoms was also controlled for. Results indicated that only smartphone location (but not smartphone power, either main effect or in interaction with location) affected performance on two tasks: OSpan (as in the first experiment) and a Go/No-Go task (a measure of inhibitory control^29^). These results leave open the question of whether the cognitive interference effect of smartphone is due to its mere presence or its availability (the possibility of receiving notifications).

Recent research also suggests that the effect of smartphone presence/availability during cognitive performance is moderated by mobile phone (psychological) dependence^21^, Internet use/attachment^30^, and nomophobia (i.e., the fear of being without access to one’s cell phone^16^), all of these constructs being assessed with self-report scales. Thus, excessive or “addicted” smartphone users are more vulnerable to the described cognitive interference effect when a smartphone is present. It is thus crucial to identify the individual differences that may moderate this cognitive interference effect. Some individuals are able to regulate their smartphone use and to ignore its presence when required^31^, whereas others struggle to not routinely engage with their device. For example, a recent study found that low self-control capacity is associated with immediate responses to smartphone signals among a sample of college students^32^. Given that successfully regulating one’s use of the smartphone (and signals) requires the ability to inhibit smartphone checking, impulsive individuals are more likely to display unregulated smartphone use. Previous research has shown that a heightened impulsivity trait is a risk factor for problematic smartphone use (for a review, see^33^). For example, excessive and uncontrolled mobile phone use was linked to low premeditation (i.e., the tendency to act without adequate consideration of potential outcomes or planning), heightened urgency (i.e., the tendency to act rashly in intense emotional contexts), and low perseverance (i.e., the inability to remain focused on boring or difficult tasks)^34–39^. Among impulsivity facets, urgency was shown to be the most strongly related to mobile phone addictive use and related negative consequences^35,36^. The urgency facet of impulsivity is composed of two related yet separable dimensions: positive and negative urgency^40^. The first refers to rash actions in response to positive emotional states (e.g., joy, euphoria), whereas the second refers impulsive actions in response to negative emotional states (e.g., sadness, fear, anger). Positive and negative urgency are affect-related impulsivity traits that play a crucial role in addictive-like behaviours^41–44^ and also constitute strong trans-diagnostic predictors of psychopathological symptoms (for a meta-analysis, see^45^). Crucially, both behavioural and neuroscience studies revealed associations between heightened positive and negative urgency traits with poorer inhibitory control^46–49^, which could at least partly explain the robust association between high urgency and excessive smartphone use. Along the same lines, a diminished ability to inhibit prepotent response inhibition has been linked to excessive smartphone use^50^. According to some scholars, existing evidence calls for more research to better specify the distinct effect of positive and negative urgency on excessive smartphone use^36^.

## The Current Research

The present study aimed to contribute to the emerging field of personality characteristics by testing whether individual differences in emotion-related impulsivity traits (positive urgency and negative urgency) moderate the cognitive interference effect of smartphone availability. Accordingly, we designed an experiment in which participants were instructed to complete a cognitive task that assessed working memory under three distinct conditions that varied in terms of smartphone availability: (i) a condition in which the smartphone is visible but turned off (low availability), (ii) a condition in which the smartphone is visible but turned on in silent mode (high availability), and (iii) a control condition in which the smartphone is not available and is replaced by a calculator (no availability). To test how smartphone availability and impulsivity conjointly impact on cognitive functioning (i.e., the cognitive interference effect), we decided to use a task that measured visual working memory (VWM) capacity. VWM is an important component of the cognitive system responsible for the maintenance and manipulation of a limited amount of visual information within a short period. VWM capacity is known to correlate with some important aspects of human cognition, such as visual attention and fluid intelligence^51^. To assess individual capacity of VWM, we used a single-probe recognition memory task^52^. In this paradigm, participants are briefly presented with a variable number (henceforth, set size) of simple visual items. After a short interval, a square appears, and participants are asked to report the presence or absence of this square among the previously memorized stimuli. Data provided by this task can estimate with a high degree of precision the average number of memory items correctly retained by a participant for each set size, namely, the individual’s VWM capacity. Given the sensitivity of this measure, we reasoned that it is a relevant variable to test the postulated cognitive interference effect. Indeed, if performance on the VWM task is lower in a specific experimental condition, this would mean that the difference observed can be linked to a cognitive interference effect of smartphone availability. We predicted that the VWM capacity of individuals characterized by elevated positive urgency would be more affected by their smartphone availability during the task (H1). We also predicted that the VWM capacity of individuals characterized by elevated negative urgency would be more affected by their smartphone availability during the task (H2). Crucially, the present study has the potential to shed new light on the interplay between technological devices, cognitive variables, and personality variables^53^ and thus contribute to extending our knowledge about the potential effect of smartphone use on human behaviour.

## Methods

This experimental study involved collecting data in a laboratory environment. Volunteer college students performed a laboratory task (in one of three different conditions; see below) and completed two self-reported questionnaires. More precisely, VWM capacity was measured by means of a single-probe recognition memory task. Individual differences in emotion-related impulsivity traits (positive urgency and negative urgency) and problematic mobile phone use were measured with validated self-reported scales. Finally, post-task questionnaires were used to check participants’ smartphone-related thoughts during the experiment. All measures were collected and manipulation was conducted in the context of the present study; no additional measures or manipulations were administered. Data and R scripts are available on the Open Science Framework: https://osf.io/8pmrx/.

### Participants

One hundred thirty-two college students (ages 18–25 years) were recruited through advertisements (community, campus, and social media) that mentioned the general objective of the study (i.e., testing the effect of smartphone availability on cognitive performance) and the inclusion criteria. The inclusion criteria were (i) being at least 18 years old, (ii) speaking Italian fluently, and (iii) owning a smartphone. Eight participants were a priori excluded: two participants reported colour recognition problems, three participants’ smartphones lit up during the task, and in three cases, the performance was not distinguishable from chance (less than 60% correct answers^54^). In addition, four participants were excluded because they did not complete the questionnaires. Thus, the final sample comprised 120 participants (65% females, *M*_*age*_ = 22.73; *SD*_*age*_ = 1.67). The institutional review board at the University of Padova gave ethical approval for the study (protocol number: 2432). Participants were informed about the details of the study and gave their informed consent. Participation in the present study was voluntary and confidential, and participants obtained no compensation. All methods used in all experiments performed during the present study were performed in accordance with the relevant guidelines and regulations of the research ethics committee.

### Procedure

Participants were individually tested in a quiet laboratory. To prevent individual differences in task performance, we systematically conducted the experiment between 9:00 am and 1:30 pm^55–57^. At the beginning of the experiment, participants provided informed consent and were asked to remove their watches during testing. We manipulated smartphone availability by randomly assigning participants to one of the three conditions: (1) turned-off smartphone (low availability), (2) silent smartphone (high availability), and (3) calculator (no availability, control condition). Participants in the turned-off smartphone condition were instructed to (i) place their smartphones face down in a designated location on the desk (consistent with Kemps *et al*.^58^) and (ii) turn off their smartphones. Participants in the silent-mode smartphone condition were instructed to (i) completely turn their devices to silent (turn off the ring and vibration) and (ii) place their smartphones face down in a designated location on the desk. Participants in the calculator condition were instructed to (i) completely turn off their smartphones and (ii) keep their phones in their bags or jackets placed out of sight (a calculator of approximately the same dimension as a smartphone was placed in the identical designated location on the desk used in the other conditions). The single-probe recognition memory task^52^ (see description below) was administrated directly following these instructions. The experimenter began the computerized task and asked the participant to follow on-screen instructions (the experimenter left the room during the session test). On completion of the task, the participants were invited to answer three questions about their task performance and about their beliefs regarding the connection between smartphones and their task performance. Next, participants completed the Short UPPS-P Impulsive Behavior Scale^59^ (Italian version^60^) and the Smartphone Addiction Inventory (SPAI)^61^ (Italian version^62^). Participants were debriefed and did not receive a monetary incentive (or collect credits) for participating in the 40-minute experiment.

### Measures

#### Single-probe task

**Figure 1:**
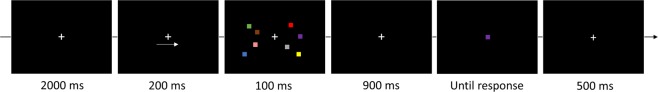
Sequences of a trial in the single-probe task.

Each trial of the task (see Fig. 1 for an example) started with a fixation dot displayed for 2000 ms, followed by an arrow lasting 200 ms that indicated the to-be-remembered subsequent hemifield (50% of cases in the left hemifield, 50% in the right). The memory array was then displayed for 100 ms and consisted of two, three, or four coloured squares in each hemifield of the screen. After a retention interval lasting 900 ms, a square was displayed at the centre of the screen until the participant responded. The participants’ task was to report the presence or absence of that square in the previously memorized array by pushing the corresponding key of the keyboard in front of them. Overall, participants completed 240 trials, divided into five blocks of 48 trials each. Before the experimental session, participants familiarized themselves with the task in a practice session (32 trials). The dependent variable was the average number of retained items for each set size as defined by the following formula: [(*K* = (hit rate + correct rejection rate – 1) × set size]. The hit rate corresponded to the mean proportion of correct responses when the probe was present in the memory array, the correct rejection rate was the mean proportion of correct responses when the probe was not present in the memory array, and the set size was the number of to-be-memorized squares (two, three, or four)^63^.

### Self-report questionnaires

The scales included in the present study were selected to prioritize instruments that have been validated in the Italian language (adapted via a traditional translation and back-translation procedure).

#### Post-task questionnaire

Thoughts after task performance were assessed by using an open-ended question (“What were you thinking during the task? Please report at least the first three thoughts”). Smartphone-related thoughts (e.g., email, text messages, calls) were recoded as 1, and smartphone-unrelated thoughts (e.g., colours, task concentration) as 0. The other two “yes” or “no” questions were as follows: “Although your smartphone was (1) turned off or (2) in silent mode or (3) inside your bag, did you expect feedback from it (rings or vibration) during the computer task?” In addition, “although the smartphone was (1) turned off or (2) in silent mode or (3) inside your bag, did you feel interrupted/bothered by your smartphone during the computer task?

#### Positive urgency and negative urgency

Positive urgency and negative urgency were assessed by using two subscales (four items for each subscale) of the Short UPPS-P Scale^59^ (Italian version^60^). Response options ranged from “strongly agree” (1) to “strongly disagree” (4). The scale was scored such that higher mean scores indicated higher levels of urgency (positive and negative). Cronbach’s alpha was 0.75 (95% confidence interval [CI] [0.66, 0.81]) for positive urgency and 0.80 (95% CI [0.73, 0.85]) for negative urgency.

#### Problematic smartphone use

We used the 26-item SPAI^61^ (Italian version^62^), which assesses the negative aspects of excessive smartphone use (e.g., negative consequences, withdrawal, compulsive behaviour, and tolerance). Response options ranged from “strongly disagree” (1) to “strongly agree” (4). The scale was scored such that higher mean scores indicated higher levels of problematic smartphone use. Cronbach’s alpha was 0.90 (95% CI [0.88, 0.93]).

### Statistical Analyses

Analyses were run by using the lme4^64^ and lmerTest^65^ packages in R. A series of linear mixed-effects models (LMMs) that used maximum likelihood t- and F-tests (Satterthwaite approximations for pooled degrees of freedom) were conducted to assess the effect of smartphone presence on VWM capacity (Cowan’s K). The full models included the fixed effects of condition; positive urgency and negative urgency, as well as their interaction; problematic smartphone use (SPAI score) as a control variable; and participants as a random effect. To test whether the cognitive interference effect of smartphone availability was greater when the task was more demanding (i.e., when a greater number of squares had to be remembered), we also ran additional models that included set sizes (two, three, or four squares) (see online supplemental materials for the detailed results of the alternative models). Post hoc tests of significant interactions (*p* < 0.05) were conducted by using the “testiInteractions” function in the R “phia” package^66^. The best-fitting model was selected by using (i) the Akaike information criterion (AIC), the lowest AIC value indicating the best-fitting model (i.e., best trade-off between goodness of fit and parsimony in terms of the number of parameters)^67^, and (ii) the Akaike model weights^68^, an estimate of the probability that a model will make the best prediction for new data, conditional on the set of models considered^69,70^. In this situation, the model with the smallest AIC and the highest AIC weight is considered to be the most plausible. Finally, effect sizes were calculated via the Psychometrica online calculator^71^.

## Results

### Preliminary analyses

To allow interpretation of the LMMs, it was necessary for us to ascertain that (i) there were no differences in the participants’ characteristics at baseline across groups and (ii) there was no sign of multicollinearity. Table 1 summarizes the participant characteristics of the three groups at baseline. The three groups did not differ in terms of age, education, and gender distribution, nor regarding negative urgency and positive urgency, problematic smartphone use, and smartphone-related thoughts. Thus, the three groups can be considered homogeneous at baseline.

**Table 1:** Sample characteristics.

	Turned off (n = 40)	Silent mode (n = 41)	Calculator (n = 39)	Statistical test
*Demographics*				
Gender (Females)	70%	61%	64%	χ^2^_(2)_ = 0.74, *ns*
Mean Age (SD)	22.60 (1.86)	22.90 (1.48)	22.69 (1.67)	F_(2,117)_ = 0.35, *ns*
Mean Education (SD)*	15.85 (1.87)	15.63 (1.80)	15.46 (1.93)	F_(2,117)_ = 0.43, *ns*
*Self-report scales/questions*				
Mean Positive urgency (SD)	2.36 (0.47)	2.33 (0.64)	2.29 (0.56)	F_(2,117)_ = 0.17, *ns*
Mean Negative urgency (SD)	2.26 (0.44)	2.29 (0.73)	2.10 (0.63)	F_(2,117)_ = 1.13, *ns*
Mean SPAI (SD)	1.62 (0.40)	1.69 (0.45)	1.60 (0.43)	F_(2,117)_ = 0.44, *ns*
SRU	15%	19%	5%	χ^2^_(2)_ = 3.70, *ns*
Expectancy#	5%	10%	3%	χ^2^_(2)_ = 1.96, *ns*
Feelings§	10%	7%	0%	χ^2^_(2)_ = 3.80, *ns*

*Number of years of education completed; SPAI = Smartphone Addiction Inventory; SRU = smartphone-related thoughts; # expecting feedback from own smartphone (rings or vibrates) during the computer task; § feeling interrupted/bothered by the smartphone during the computer task.

Regarding the multicollinearity check, we considered the magnitude of correlation coefficients and computed the variation inflation factor (VIF) and tolerance indices. The magnitude of correlation coefficients was relatively modest, ranging from 0.28 to 0.59. In particular, positive correlations were observed between negative urgency and positive urgency (*r* = 0.59; *p* < 0.001), negative urgency and SPAI (*r* = 0.28; *p* = 0.002), and positive urgency and SPAI (*r* = 0.38; *p* < 0.001). Consequently, correlations were not high enough to raise concern about collinearity. No multicollinearity issues were detected for the LMMs. All predictors had tolerance values of at least 0.60 and VIF values below 1.67. Tolerance values greater than 0.02 and under 2.5 for VIF were considered reliable cut-off points for the absence of multicollinearity^72^. Finally, the average value of K scores was 2.23 (SD = 0.58; skewness = 0.03, kurtosis = 0.01).

### Effects of Smartphone Availability and Impulsivity Traits on VWM Capacity

**Figure 2:**
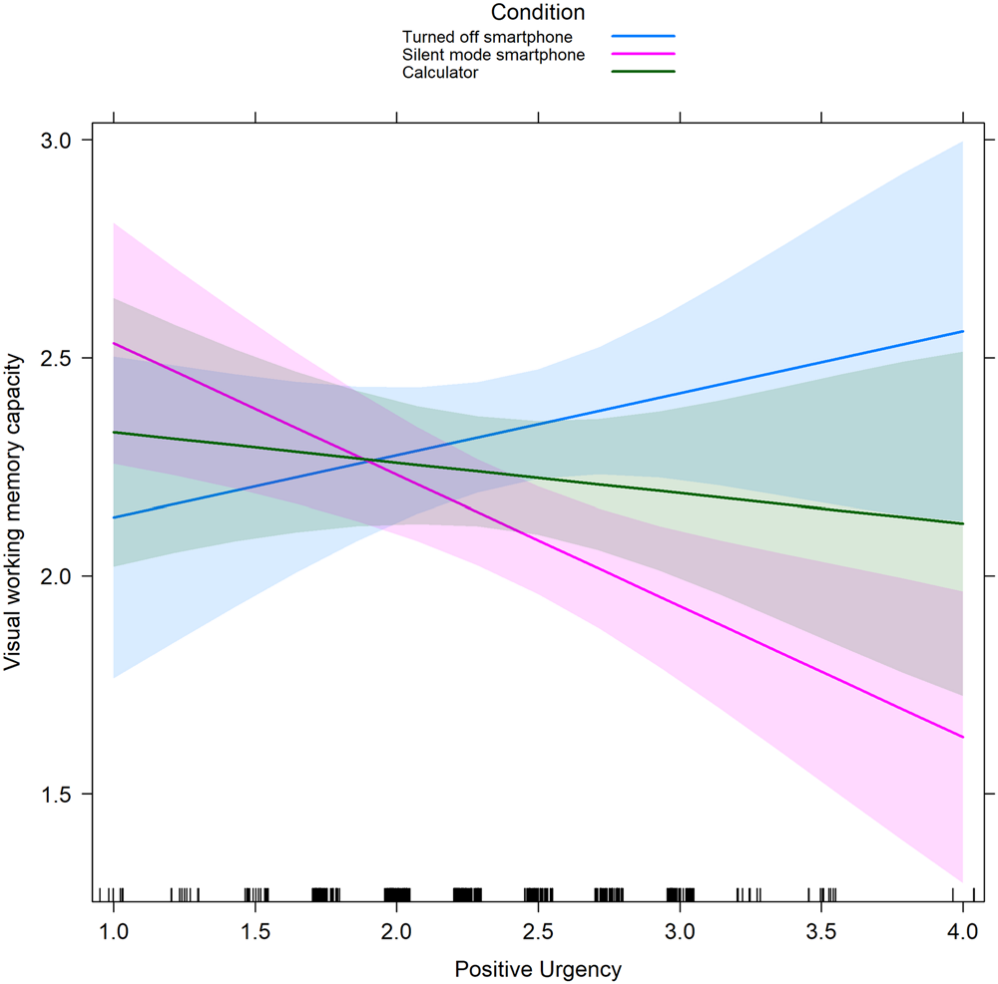
Interaction plot for positive urgency and condition in relation to visual working memory capacity. Confidence intervals of 95% are presented in blue/pink/green.

To test how participants with higher emotion-related impulsivity traits (positive urgency and negative urgency) performed in the task in terms of smartphone availability, we computed and compared the following models: the null model with intercept only and no predictors (M0); a model with positive (or negative) urgency and problematic smartphone use (M1); a model with condition and problematic smartphone use (M2); a model with condition, positive (or negative) urgency, and problematic smartphone use (M3); and a model with condition and positive (or negative) urgency, their interaction, and problematic smartphone use (M4). Table 2 shows the results for the model comparisons separately for negative urgency and positive urgency. As the AIC selection method indicated, the model with the condition × positive urgency interaction term (M4) best fitted the data (Table 2), with a probability of being the best of 0.59. Regarding this model (M4), problematic smartphone use (SPAI score, *χ*^2^ *(1)* = 0.68, *p* = 0.40) and positive urgency (*χ*^2^ *(1)* = 1.62, *p* = 0.20) were not significantly associated with task performance. Although condition showed a tendency toward significance (*χ*^2^ *(*2*)* = 5.12, *p* = 0.07, *d* = 0.42), a closer inspection of the results for condition indicated that participants performed worse in the silent-mode smartphone condition (*B* = 0.78, 95% CI [0.15, 1.41], *t* = 2.05, *p* = 0.03, *d* = 0.38) than did participants in the turned-off smartphone condition, whereas no differences were found between participants in the calculator condition and those in the turned-off smartphone condition (*p* = 0.32) or between participants in the calculator condition and those in the silent-mode smartphone condition (*p* = 0.22). There was an interactive effect of condition × positive urgency (*χ*^2^ *(*2*)* = 7.22, *p* = 0.03, *d* = 0.51), indicating that the effects of smartphone presence on VWM capacity were moderated by individual differences in positive urgency (see Fig. 2).

**Table 2:** The AIC model comparison analysis (for negative urgency and positive urgency separately).

Model	Fixed effects	AIC	Akaike weight
M0	~1	619.74	0.09
M1	NU + SPAI	618.34	0.18
M2	Condition + SPAI	617.19	0.32
M3	Condition + NU + SPAI	617.90	0.22
M4	Condition + NU + SPAI + Condition × NU	618.52	0.17
M0	~1	619.74	0.04
M1	PU + SPAI	618.31	0.09
M2	Condition + SPAI	617.19	0.16
M3	Condition + PU + SPAI	617.57	0.13
M4	Condition + PU + SPAI + Condition × PU	614.56	0.59

All models with participant as a random effect; NU = negative urgency; PU = positive urgency; SPAI = Smartphone Addiction Inventory; AIC = Akaike information criterion.

Contrast analyses showed that participants who tended to act rashly in response to extremely positive moods had poorer task performance in the silent-mode smartphone condition than did participants in the turned-off smartphone condition (*χ*^2^ *(1*) = 6.42, *p* = 0.03, *d* = 0.49). Therefore, individuals with higher positive urgency are negatively affected by the high availability of their smartphone during the VWM task. No differences were found among participants in the turned-off smartphone condition versus the calculator condition (*χ*^*2*^ *(1)* = 1.28, *p* = 0.27), or in the silent-mode smartphone condition versus the calculator condition (*χ*^*2*^ *(1)* = 2.51, *p* = 0.24).

Regarding task complexity (see the online supplemental material for details), results showed that the effect of positive urgency in interaction with condition (silent-mode smartphone condition vs. turned-off smartphone condition) on task performance was statistically significant only in the most difficult part of the task (*set size* = 4) (*F*_*(1,114)*_ = 8.36, *p* = 0.01), but not for *set size* = 3 (*p* = 0.08) or *set size* = 2 (*p* = 0.07).

## Discussion

The main aim of the current study was to test the influence of positive urgency and negative urgency (two emotion-laden impulsivity traits constituting the trans-diagnostic factors of psychopathology) on the cognitive interference effect of smartphone availability by using an experiment in which participants were exposed to various degrees of availability of their smartphones (high availability, low availability, no availability). The present study revealed that positive urgency contributes to the differential effect of smartphone availability on cognitive performances as assessed with a task involving VWM capacity and attentional processes. The discussion is divided into two parts. The first part pertains to the effect of smartphone availability on VWM capacity, and the second part concerns the moderating role of emotion-laden impulsivity traits in this effect.

The current study partially replicates previous research in the field^21,22^ by emphasizing the multiplicative effect of smartphone location and power (“on” and “off”) as cognitive interference for participants. More specifically, we found that smartphone availability had a detrimental effect on task performance only when these devices were turned on (in silent mode, high availability) and not when they were turned off (low availability). It is thus possible that impaired performance in the task used to assess attentional and VWM capacities is related to incoming notifications (or the possibility of receiving a notification), implying that participants in the silent-mode smartphone condition were perturbed by possible missed notifications or messages. It is worth noting that this effect could be promoted by psychological processes not measured in the present study, such as the fear of missing out^73^. It is plausible that smartphones in silent mode might promote cognitive interference in an automatic way (i.e., without conscious monitoring) given that participants in the silent-mode smartphone condition did not report more smartphone-related thoughts in the post-task questionnaire (see Measures section) than did participants in the other two conditions. Notably, the silent-mode smartphone (high availability) and the calculator (no availability) conditions similarly affected performances in the working memory task. It is thus likely that the calculator condition was not an ideal control condition, as totally restricting access to the smartphone might have perturbed or distracted the participants, instead of modelling a situation in which the smartphone was not available. Indeed, previous studies showed that adolescents and young adults have a strong feeling of dependence on their mobile phone^74^ and that these technological devices are even stronger than natural reinforcers such as food^75^. It is thus possible that the calculator condition is not directly comparable to the other two conditions (which differ only in terms of availability), which makes the interpretation of the results related to this condition uncertain. From the current unexpected findings obtained in the calculator condition, we suggest that future studies not use it as a control condition.

The present study shows that positive urgency plays a role in explaining the differential effect of smartphone availability on VWM capacity. More precisely, participants with higher positive urgency had poorer task performance in the silent-mode smartphone condition (high availability) than did participants in the turned-off smartphone condition (low availability). Considering that positive urgency has notably been related to increased difficulty in inhibiting prepotent responses^47^, it might be that individuals with high positive urgency have more difficulty in inhibiting mobile phone use when it is available, as mobile phone use (e.g., checking email or notifications) involves highly automatized habits^76,77^. As previous research showed that smartphone reinforcing efficacy is associated with positive (reward related) use motives^75^, the specific effect of positive urgency found in the present study might be because the cognitive interference effect of a smartphone is more pronounced when one is experiencing or anticipating positive affect. Indeed, it is well established that positive affect and emotional arousal interfere with cognitive control (e.g., the increase of interference and distractibility^78,79^). Thus, H1 was supported. In the present study, the cognitive interference effect of smartphone availability, in combination with higher positive urgency, was largest for the more difficult part of the memory task (four to-be-remembered squares), which is consistent with the results of previous studies that found heightened cognitive interference in situations characterized by a greater demand on attentional and cognitive resources^22,80^.

Inconsistent with H2, negative urgency (which was also related to prepotent inhibition difficulties in previous studies^46,47^) did not interact with the various conditions of our experiment. The existing literature highlights the link between negative urgency and problematic smartphone use;^35,36^ however, no study to our knowledge has examined the effect of negative urgency on VWM capacity in conditions whereby smartphone availability is manipulated, which is different from the problematic behaviours targeted in previous studies (with self-reports)^36^. It is possible that individuals with high negative urgency display impaired response inhibition related to smartphone availability only in emotional contexts in which negative affect is experienced, or during the inhibition of negative stimuli^81^. In relation to this, two recent studies found that trait negative urgency is associated with more rash behaviour following a stressful experience^82,83^, which was not the case in the present study (our participants were not experimentally stressed). Thus, the implication of emotional contexts (induced moods) or stressful experiences (stress induction) should be subjected to further testing. Another potential explanation for this differential effect of positive and negative urgency relates to the individual characteristics of our sample of college students. A recent meta-analysis^45^ found that the effect size for negative urgency with response inhibition was robust in a clinical sample (weighted mean *r* = . 34) but was very small in a student and a community sample (weighted mean *r* = . 12). It is thus likely that a potential effect of negative urgency on smartphone interference would appear only in clinical samples or in samples of individuals with marked psychopathological symptoms. In support of that view, recent research found that negative urgency is associated with problematic smartphone use^36^, showing that this risky behaviour is more likely to occur in the context of intense negative emotions among people who are characterized by post-traumatic stress disorder symptoms.

Some limitations of the study have to be acknowledged. First, we used a convenience sample of college students, which hinders the generalizability of our findings to other groups (e.g., older people). Yet, this population was considered of primary interest for our study because college students (i) are digital natives likely to be heavy smartphone users, (ii) are liable to experience negative academic consequences because of hazardous smartphone use, and (iii) have been shown to constitute an at-risk population regarding excessive and addictive smartphone use^1–4^. Second, we relied on self-reported measures, which are known to be at least partly flawed, especially when it comes to potentially stigmatized behaviours such as excessive mobile phone use. Third, the study did not manipulate emotional states (e.g., via induction) or measure actual affect states, implying that our explanations regarding the differential effect of positive and negative urgency remain tentative. Fourth, although we focused on emotion-laden impulsivity traits as primary moderators, it is possible that other factors, such as specific psychological factors (e.g., fear of missing out or “FOMO”^84^), moderate the cognitive interference effect in various ways. Fifth, although we made all our material and data open access via the Open Science Framework, we did not preregister the study hypotheses. Future studies that use preregistration are thus suggested in order to confirm and extend the present findings.

Despite these limitations, the present study adds to previous research by improving our knowledge about how smartphone availability competes with high-level cognitive processes and thus creates a cognitive interference effect. The cognitive interference generated by the simple availability of a smartphone may have implications in various contexts and situations (e.g., driving, performance in school, and productivity in the workplace). In terms of implications, our results call for considering regulations or information campaigns that emphasize the merits of turning off (when possible) the smartphone in specific contexts, such as while driving or when in classrooms. Moreover, clarifying the role of emotion-related impulsivity in smartphone interference might be of value for practitioners in order to build specific educational university-based programs on new technology aimed at tackling unregulated smartphone use, as well as at promoting the positive use of smartphones and emotional skills (e.g., mindfulness-based group interventions to reduce urgency-related behaviours in response to positive emotional states).

## Supplementary information

Supplementary analyses

## Data Availability

Data and scripts are available on the Open Science Framework: https://osf.io/8pmrx/.
