# Supplementary material for: Emotion-related impulsivity moderates the cognitive interference effect of smartphone availability on working memory
Source: Sci Rep. 2019 Dec 6;9:18519. doi: 10.1038/s41598-019-54911-7 (PMC6898282; doi:10.1038/s41598-019-54911-7)
Supplement: Supplementary file 1 — Supplementary analyses [file 41598_2019_54911_MOESM1_ESM.pdf]

Emotion-related impulsivity moderates the cognitive interference effect of smartphone availability on working memory

Natale Canale[1,*], Alessio Vieno[1], Mattia Doro[1], Erika Rosa Mineo[1], Claudia Marino[1] and Joël Billieux[2]

[1] Department of Developmental and Social Psychology, University of Padova, Padova, Italy
[2] Addictive and Compulsive Behaviours Lab, Institute for Health and Behaviour, University of Luxembourg, Esch-sur-Alzette, Luxembourg
*natale.canale@unipd.it

As indicated by the AIC selection method (table S1), the model with the condition × positive urgency × setsize interaction term (M5) best fitted the data (Table S1).

Models	Fixed Effects	AIC	Akaike weight
M0	~ 1	619.74	<.001
M1	Condition + SPAI+Setsize	340.58	0.15
M2	Condition +PU+SPAI+Setsize	340.96	0.13
M3	Condition + SPAI+PU*Setsize	341.88	0.08
M4	PU+Spai+Condition*Setsize	343.96	0.03
M5	SPAI+Condition+PU+ PU*Setsize+ Condition*Setsize + Condition*Setsize*PU	337.89	0.60

PU = positive urgency; Set Sizes = two, three, four squares; SPAI = Smartphone Addiction Inventory

Table S1. The AIC model comparison analysis

With regard to M5 (table S2), there was an interactive effect of condition × positive urgency × setsize ($\chi2$ *(4)* = 11.53, *p* = 0.02).

Coeffic	χ^2	df	*P* values
Condition	4.82	2	.08
Positive Urgency	1.62	1	.20
Spai	0.68	1	.40
SetSize	550.12	2	<.001
Condition X PositiveUrgency	6.80	2	.03
Condition X SetSize	5.17	4	.26
PositiveUrgency X SetSize	3.22	2	.20
ConditionXPositiveUrgencyXSetSize	11.53	4	.02

Table S2. M5 model

Figure S1. Interaction plot for positive urgency, Set Sizes (2, 3, 4 squares) and condition in relation to visual working memory capacity. Confidence intervals of 95% are presented in blue/pink/green

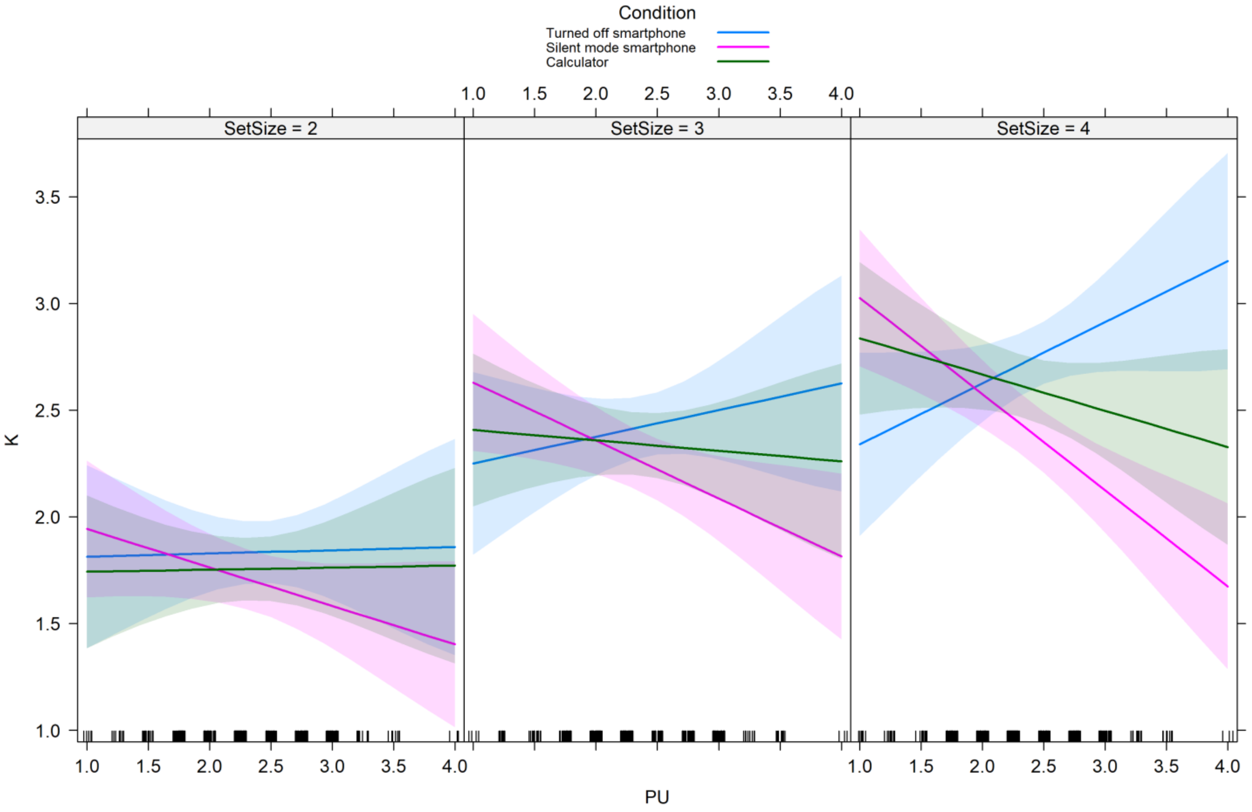

.
